# Excerpta

**Published:** 1880-02

**Authors:** 


### EXCERPTA.

TRANSMISSION OF HYDROPHOBIA FROM MAN TO RABBITS.—M. Raynaud inoculated a number of rabbits with the saliva and blood of a hydrophobic patient on the day before death. The inoculations of blood gave negative results; the inoculations of saliva, however, were followed by rabies in a relatively short space of time, at most a few days. M. Raynaud also extirpated the submaxillary glands of two rabid rabbits and inocolated them on two healthy rabbits; the results were equally positive, that is to say, the inoculations were followed in a short time by the symptoms of rabies. No reliable case of the transmission of rabies from man to man has yet been recorded, but M. Raynaud thinks that after his demonstration of the possibility of its transmission from man to rabbits, it is fair to conclude that the results would be identical in the case of man if accidentally inoculated.—*Le Progrès Médical, Nov.* 15, 1879. *N. Y. Med. Record.*

THE NATIONAL BOARD OF HEALTH.—Exception has been taken to the editorial in the last issue which stated that the "board had been composed mostly of general practitioners, residing at such long distances from one another that mutual acquaintance is impossible, that frequent meetings are impracticable, and that the duties of a board so organized must necessarily be delegated in a large degree to one or more persons mutually acquainted and located at some central point." Subsequent inquiry has shown that the board as a whole has met once a month since June, that four or five of its members have met weekly, and that the distant members of the board have generally attended these meetings at great personal sacrifice of time and money, amounting, as one member reports in his own case, to over a thousand dollars in six months. It thus appears that the meetings have been more fully attended and more frequent than was at first supposed; but since these frequent and full meetings appear to depend upon the inclination of the distant members to sacrifice, in time and money, two thousand dollars a year each to the interests of national sanitation, it would appear the duties of this board must sooner or later be delegated to a few, and it is natural to suppose that these few would have "mutual acquaintance" and a "central location." The questions of local sanitation and urban cleanliness are the only ones which could not properly have been referred to the Marine Hospital service. The usefulness or uselessness of the board therefore rests upon its ability or inability to deal with these questions more efficiently than the local boards. Under its present organization and by its present methods the board may succeed beyond the expectation of the profession, but even then the question will arise whether all of its success could not have been attained with more economy and with less hurrah under the management of the Marine Hospital service.—*Chicago Med. Gazette.*

MEDICAL CHARITY AND WHAT COMES OF IT.—"Having, in a long time of practice, both from choice and necessity, done a great deal of gratuitous service, I have yet to find a single case where my charity work was appreciated. Those who pay nothing always offset it by liberal abuse, which keeps away those who would pay. Your charity case may be a worthy man, but if you were making a struggle to build a house, would he work for you at reduced rates (or for nothing)? It is the doctors themselves, who allow their kind feelings to overrun their judgment, that are responsible for this wholesale robbing to which every doctor in this land is subjected. We deal with the most afflicted; so does the undertaker, who is not expected to work for nothing. We can maintain no rights that we weakly yield to extortion.

"The doctors are most universally regarded as rich persons who ride about for exercise, and practice for philanthropy, to be paid if everything turns out lovely; if not, they can go to the d—l, and must not complain. The people who pay are always grateful; the thieves are like other dead-beats, abusive, and always most exacting and querulous. . . . If the patient cannot pay for what might save his life, his friends or the public should. It is easier for the town to shoulder the cost than two or three poor devils who had the bad luck to study physic. Now or never is the time to put ourselves on the same footing with other businesses, and, as we have the same losses, we must ask for the same gains."—*Canada Lancet.*

NEW METHOD OF PRESERVING DEAD BODIES.—The German government has recently bought the patent for a new preservative fluid. It is claimed for it that the bodies, even after years, retain their color, form and flexibility. Decay is entirely prevented, and the muscles even keep their natural color.

The bodies are saturated in a liquid made as follows:

| | |
|---|---|
| ℞.—Alum . . . . . | 100 |
| Sodii Chlorid. . . . . . | 25 |
| Potas. Nitrat. . . . . . | 12 |
| Potas. Carb. . . . . . | 60 |
| Acid. Arsenici. . . . . . | 10 |
| Aqua . . . . . . | 1000 |

This solution is cooled and filtered. There are then added to ten litres of the fluid four litres of glycerine and one litre of methylic alcohol. From two to five litres of the liquid are used in saturating the body to be preserved.—*N. Y. Med. Record.*

THE following is a valuable remedy for scanty, irregular menstruation, when associated with and dependent upon anemia, neuralgia and neurasthenia:

| | | |
|---|---|---|
| ℞.—Tincture Ferri. Chl. | . . . | ʒ x. |
| Liquor Potassa Arsenitis | . . . | ʒ ij. |

*M. S.* Gtt. xij after each meal, through a glass tube, in about one-third glass of water.

The following prescription is specially serviceable in the various nervous manifestations which often accompany the menopause:

| | | |
|---|---|---|
| ℞.—Sodii Bromidi | . . . | ʒ iv. |
| Tincture Nucis Vomica | . . . | ʒ ij. |
| Elixir Calisaya | . . . . | ℥ ij. |
| Syrupi Pruni Virg. | . . . | ℥ i. |
| Elixir Simplicis | . . . . | ʒ vi. |

*M. S.* ʒ ij two, three or four times a day, as needed.

—*Chicago Med. Gazette.*

CHILDBIRTH AFTER OVARIOTOMY.—On the 29th of June, 1875, the late Dr. Washington L. Atlee, assisted by his son, Dr. Atlee, removed a multilocular ovarian tumor from Mrs. J. C., of this city.

Although the patient at the time seemed very much reduced, and the weather was extremely warm, she made a rapid and excellent recovery, being able to sit up in a rocking-chair for several hours on the ninth day.

On the 21st of June, 1877, after a difficult and tedious labor (occipito-posterior presentation), I delivered her with the forceps of a fine, large boy, weighing eleven pounds avoirdupois. Subsequently, on the 4th of October, 1879, I again delivered her of a daughter weighing between eleven and twelve pounds.

This case was one of breach presentation, the labor not being more than ordinarily difficult for so large a child.

Although pregnancy and delivery at full term under these circumstances are not of unusual occurrence, yet, as Dr. J. Ewing Mears remarks (*Medical Times*, August 16, 1879), the cases are sufficiently important to be placed on record.

JOHN F. WALKER, M. D.

201 Columbia Avenue, Philadelphia. —*Phila. Med. Times.*

DIARRHŒA A ZYMOTIC DISEASE.—In my yearly report and tables, forwarded to you, you have noticed that my returns of diarrhœa and the Registrar-General's do not correspond. You are aware that in all printed tables, diarrhœa is classed among the zymotic diseases; now, I think it very unfair to the district, and calculated to give a very false impression of its sanitary condition, to swell its rate per 1000 of zymotics by the admission of cases of diarrhœa, in many cases certainly dependent on intestinal irritation from dentition, and even in summer diarrhœa, probably due more to this cause than any unsanitary condition of the district in which they occur. The diarrhœa of adults, too, in warm weather, is usually more due to climatic change and unwholesome fruit than any other cause. I think, then, such cases should be referred to their respective headings of "infantile diseases of dentition," and "diseases of digestive system." Of course, care should always be taken in investigating any such cases, that no condition of water contamination or drain infection be overlooked, as, no doubt, over and above the causes given, summer diarrhœa is largely affected by such causes; still, you will see I contend for the principle that this is not invariably the case, and that the assumption that it is so is unfair; though, of course, I am open to conviction if I am proved to have overlooked anything in the argument.

J. MAY, Jun., M. O. H., Devonport.

[Opinion is somewhat divided as to the classification of diarrhœa as a zymotic disease, although the greatest epidemiological authorities argue that infantile summer diarrhœa is of zymotic origin. Many deaths, especially of adults, are certified as the result of diarrhœa, which are really due to organic disease, but it is next to impossible to separate them from the rest of the deaths referred to diarrhœa. The great object to be aimed at in the publication of local mortality statistics, for comparative purposes, is to secure uniformity, and thus it becomes important not to depart from the Registrar-General's practice of including diarrhœa as one of the seven principal zymotic diseases. No local injustice can be committed if all the districts are treated alike.—ED.]—*London Sanitary Record.*

TREATMENT OF HEPATIC CALCULI.—Some very positive statements on this subject are made by Dr. T. H. Buckler in the Boston *Medical and Surgical Journal.* Referring to Dr. T. G. Thomas' enumeration of the operation of cutting into the gall-bladder as one of the recent surgical triumphs, he asserts that such a procedure is unwarrantable. Cholesteric gall-stones can always be dissolved away by large doses of chloroform, especially if combined with succinate of iron. The latter agent also may alone accomplish the desired solution and effect a cure. In Dr. Buckler's last three cases, treated successfully, he gave ten drops of chloroform every four hours, and a teaspoonful of Stewart's hydrated succinate of the peroxide of iron half an hour after each meal. He has sometimes given a teaspoonful of chloroform every six hours without causing any bad symptoms to the patients, and with the result of a cure within a week.—*N. Y. Medical Journal.*

The Value of Benzoate of Soda in Diphtheria.—Dr. Grändinger (*Rundschau*, October, 1879) was induced to try benzoate of soda in diphtheria by the encomiums heaped upon it by Klebs and Letzerich. His observations were made in one of the hospitals for children in Vienna. The doses in which the medicine was administered were those recommended by Letzerich, namely, to children of one year old, 5.0 grammes; children from one to three, 7.0 to 8.0 grammes; children from three to seven, 8.0 to 10.0 grammes; children over seven, 10.0 to 15.0 grammes of benzoate of soda, dissolved in from 80 to 110 grammes of water.

Besides this internal use of the drug, the substance was dusted directly on the diseased throat two or three times a day.

The other treatment of the little patients was precisely the same as was used when other medicines, such as ice, chlorate of potash, stimulants, etc., were employed.

Of the *seventeen* patients subjected to this treatment, *eight* died.

Besides these seventeen cases, there were admitted into St. Anno Hospital, from the first of January to the 15th of June, *seventy-six* other cases of the disease who were treated in the old way—with ice, chlorate of potash and stimulants. Of these, *forty-nine* recovered and *twenty-five* died; two were still under treatment when Dr. Grändinger's paper was written. The results are very different from those of Letzerich.— *Virginia Med. Monthly.*

German Therapeutics.—New Orleans *Medical and Surgical Journal:* There is strong reason to believe that the modern treatment of disease in Germany has greatly deteriorated since the days of Niemeyer. Theorizing, histology, and diagnostic refinements have taken the place of the effort to cure disease by rational empiricism. Witness the next to worthless therapeutics in *Ziemssen's Cyclopedia.* A Vienna correspondent in the Canada *Medical and Surgical Journal* gives some striking statistics. In Bamberger's clinic, of twenty-seven cases of pneumonia seventeen died; twenty-four per cent. of all cases of typhoid fever die; facial erysipelas is "frequently fatal," etc. We do not believe that the case-book of the average American physician shows anything like this mortality, and our city hospitals certainly do not. It looks as if medical science in Germany was running to seed.

NO FACT is more evident than the highest order of physicians and surgeons are not men remarkable for their knowledge of microscopy, of experimental physiology, and the other branches of theoretical medical science; and conversely, that the microscopists and pure physiologists are not remarkable as physicians, and, indeed, cannot be. The attempt to pervert the proper purpose of medical schools, and to give a merely science aspect to medical teaching, is a fashion of the time which, if it gain more adherents, is likely to do serious mischief to the cause of medical education; for young men, allured by the glitter of scientific work, will neglect the important and really more difficult attainments of true professional studies.

DR. BARTHOLOW.

—*Louisville Medical Review.*

RESTORATION OF THE MENSES.—*Editor Med. Brief:* For the benefit of Dr. S. W. Hopkins, I give the following recipe for the restoration of the menses, when stopped by cold or exposure, which I have never known to fail:

| | | |
|---|---|---|
| ℞.— | Gum Guaiac. . . , . | ℥ ij. |
| | Balsam Canadensis . . . . | ℥ ii. |
| | Oil Sassafras . . . . . | ʒ ij. |
| | Corrosive Sublimate . . . . | grs. xx. |
| | Alcohol . . . . . | ℥ viij. |

Sig.: Twenty drops in wine glass of water night and morning, commencing ten days before expected time.

Dissolve the guaiac. and balsam in one-half of the alcohol, and corrosive sublimate in the other half; let guaiac. and balsam digest for five or six days, then pour off the clear liquid and mix with the corrosive sublimate and add the oil sassafras.

Dr. G. W. Watters will find the fluid extract viburnum prunifolium, in half teaspoonful doses, to relieve the distressing pains of dysmenorrhœa. C. HOOVER, M. D.

McCOOMBS CITY, MISS., Jan. 19, 1880.
—*St. Louis Medical Brief.*

INCONTINENCE OF URINE IN CHILDREN.—Dr. Farquharson read a paper upon this subject before the Harveian Society. After some preliminary remarks on the bearing of incontinence of urine on surgery and obstetric medicine, he referred to the subject under three headings. In some cases this affection is found in children of pale, weakly organization, depressed and languid, and feeling keenly their infirmity. Here there is, no doubt, some weakened condition of the sphincter vesicæ, or of the nervous centres in the lumbar cord; and tonic remedies, and more especially small doses of wine, will usually act with excellent effect. Secondly, there are cases of much greater severity, usually dating from a period shortly after birth; and here it is necessary to make a distinction of enuresis by day and that by night, for the latter is much more difficult of cure than the former, and frequently resists all medicinal treatment, departing, if it does so at all, spontaneously about the period of puberty. The remedies which have generally been spoken of as most deserving of confidence are those which act on unstripped muscular tissue, and of these belladonna is the only one which, in the experience of the author, has given good results. It is necessary to give full doses, and two ounces have been administered to a boy of seven before success, and even then only temporary success was attained. Ergot has proved disappointing, and santonine has been entirely without influence under the morbid condition. Class three includes those cases which may support the belief that incontinence of urine is truly a neurosis; for here we find this symptom coinciding with, and even alternating with, other nervine lesions. Thus, on two occasions it was observed concurrently with eczema, and once a very long standing case was attacked with chorea, during the continuance of which perfect control over the bladder was regained. Nervine tonics are of little use here; but the careful use of galvanism seems specially indicated, as well as blistering over the fifth lumbar vertebra, where modern experiment has shown the motor centre to be situated. The recently proposed plan of excluding meat from the dietary was found not to be of much service, no especial acidity of urine being ever observed to require the counteracting agency of purely non-nitrogenous food.—*The British Medical Journal.*

IDENTIFICATION OF THE PRINCE IMPERIAL.—The circumstances of the Prince Imperial's death have revived a question which has been somewhat neglected by lawyers and physicians, viz: the importance of the teeth as a means of identification of deceased persons. The late Prince Imperial had been so much disfigured that identification would have been extremely difficult but that the Prince had had four small cavities in the first molar teeth filled with gold by Dr. Rottenstein, of Paris, and had met with a slight accident in April, 1876, from a blow on the front teeth, which had made it necessary to file the teeth a little in order to smooth the enamel. These constituted signs which are unalterable even by ages; and, as careful dentists keep usually a record of such operations, they afford a means of identification which is unerring, and which, as in the present instance, was of great value, and might, under certain circumstances, be of the highest importance. —*British Medical Journal.*

At a meeting of the dental practitioners of Baltimore, at the Baltimore College of Dental Surgery, on Tuesday evening, January 6, 1880, the following resolutions were unanimously adopted:

Whereas, The Dental Practitioners of Baltimore city having been called together to pay a tribute of respect to the memory of the late Dr. Samuel Stockton White, of Philadelphia, whose death may be regarded as both a professional and public loss; therefore be it

*Resolved*, That the Dental Practitioners of Baltimore city have learned with deep regret of the death of one who has been for many years closely identified with the dental profession, at that period of life when the mental powers are in their fullest vigor, when his honorable career as an enterprising business man had won for him a world-wide reputation, and whose personal qualities, as exemplified in every relation of life, had secured the warm attachment and high respect of a large circle of devoted friends.

*Resolved*, That the Dental Practitioners of Baltimore city fully recognize the obligations American dentistry owes to the late Dr. S. S. White through the active agencies he has awakened for the elevation of the science, his devotion to its interests, and the zeal, energy and intelligence he displayed in useful inventions and dental literature.

*Resolved*, That this expression of appreciation of the worth of the late Dr. Samuel Stockton White, in the form of a copy of these resolutions, be transmitted to the bereaved family of the deceased, and that they also be published in the different dental journals.

Respectfully submitted,

F. J. S. Gorgas,
T. S. Waters,
A. P. Gore,
Committee.

Professors Gorgas and Winder, on the part of the Faculty of the Baltimore College of Dental Surgery, and Dr. H. H. Keech, on the part of the Dental Practitioners of Baltimore city, were appointed to attend the funeral in Philadelphia.

---

CAUSES OF THE DELAY.—The February number of our journal has been delayed longer than we expected, but the enlargement and improvement in the building and publishing facilities of the printing office consumed more time than was contemplated when the matter was prepared for the press. We have telephonic communication now with our respective offices, over two miles apart, and with the printing office also; and are therefore in condition to facilitate our editorial duties and the publication of the journal hereafter. It is our purpose to issue it regularly between the tenth and fifteenth of each month, and not on the first of the month, as originally contemplated, owing to the impossibility of getting the mortuary and thermometrical reports of the preceding month before the first of the month in which the journal is published.

### JANUARY REPORT OF DEATHS AND INTERMENTS AT BALTIMORE.

| CAUSES OF DEATH. | NO. |
|---|---|
| Abscess | 3 |
| Anemia | 1 |
| Aneurism of Aorta | 1 |
| Apoplexy | 14 |
| Asphyxia | 3 |
| Asthenia | 5 |
| Bright's Disease | 14 |
| Burns and Scalds | 4 |
| Capillary Bronchitis | 7 |
| Cancer | 15 |
| Casualties | 2 |
| Child Birth | 1 |
| Cholera Infantum | 1 |
| Cirrhosis of Liver | 3 |
| Concussion of Brain | 1 |
| Congestion of Brain | 4 |
| " Liver | 1 |
| " Lungs | 1 |
| Consumption of Lungs | 110 |
| Convulsions | 37 |
| Compression of Brain | 7 |
| Croup | 13 |
| Cyanosis | 2 |
| Congestive Chill | 1 |
| Catarrh | 1 |
| Cardiac Embolism | 1 |
| Dentition | 6 |
| Diarrhœa | 1 |
| Diphtheria | 48 |
| Disease of Heart | 24 |
| " Liver | 1 |
| " Hip | 1 |
| Dropsy, General | 7 |
| Dyspepsia | 1 |
| Dysentery | 2 |
| Empyemia | 1 |
| Erysipelas | 1 |
| Entero Colitis | 1 |
| Endocarditis | 1 |
| Fever, Intermittent | 2 |
| Fever, Puerperal | 3 |
| " Remittent | 1 |
| " Gastro Enteric | 1 |
| " Scarlet | 44 |
| " Typhoid | 18 |
| " Catarrh | 1 |
| Fracture Skull | 3 |
| Fistula in Ano | 1 |
| Gangrene, Senile | 2 |
| Gastro Enteritis | 2 |
| Hydrocephaloid | 2 |
| Hernia, Strangulated | 1 |
| Hemorrhage of Bowels | 1 |
| " Lungs | 2 |
| " Uterus | 1 |
| " Brain | 1 |
| Hæmoptysis | 1 |
| Hydrocephalus | 3 |
| Hydrothorax | 4 |
| Inanition | 2 |
| Insanity | 1 |
| Ileus | 1 |
| Inflammation of Bowels | 1 |
| " Stomach | 3 |
| " Bronchi | 10 |
| " Liver | 1 |
| " Pericardium | 3 |
| " Peritoneum | 5 |
| " Tonsils | 1 |
| Intemperance | 2 |
| Jaundice | 1 |
| Marasmus | 13 |
| Meningitis | 10 |
| " Tubercular | 4 |
| " Cerebro Spinal | 1 |
| Murder | 2 |
| Œdema of Lungs | 3 |
| Old Age | 31 |
| Paralysis | 6 |
| Pneumonia | 50 |
| " Typhoid | 4 |
| Poison | 1 |
| Premature Birth | 10 |
| Puerperal Metritis | 2 |
| " Peritonitis | 1 |
| Pyemia | 1 |
| Progressive Locomotor Ataxia | 1 |
| Pulmonary Engorgement | 1 |
| Rheumatism, Inflammatory | 1 |
| Rupture | 1 |
| Scrofula | 6 |
| Spinal Bipida | 1 |

### MORTUARY REPORT—(CONTINUED).

| CAUSES OF DEATH. | NO. | CAUSES OF DEATH. | NO. |
|---|---|---|---|
| Softening of Brain | 2 | Uremia | 2 |
| Septicemia | 2 | Unknown Infantile | 2 |
| Stricture, Intestines | 1 | Vesicular Emphysema | 1 |
| Syncope | 2 | Whooping Cough | 5 |
| Suicide | 1 | | |
| Syphilis | 3 | Totals | 652 |
| Tetanus Neanatorus | 1 | | |
| Trismus Nascentium | 2 | Annual Death Rate per 1000 during the Month | 18.86 |
| Tumors | 3 | | |
| Ulcers | 2 | | |

### THE DEATHS REGISTERED IN THE MONTH INCLUDE

| | | | |
|---|---|---|---|
| Under 1 year | 130 | Between 30 and 40 years | 55 |
| Between 1 and 2 years | 43 | " 40 and 50 " | 50 |
| " 2 and 5 " | 63 | " 50 and 60 " | 49 |
| | | " 60 and 70 " | 58 |
| Under 5 years | 236 | " 70 and 80 " | 45 |
| Between 5 and 10 years | 51 | " 80 and 90 " | 15 |
| " 10 and 15 " | 9 | " 90 and 100 " | 6 |
| " 15 and 20 " | 20 | " 100 and 110 " | 3 |
| " 20 and 30 " | 55 | Above 110 years | |

### NATIVITY, ETC.

| NATIVITY. | MALES. | FEMALES. | TOTALS. |
|---|---|---|---|
| United States (White) | 181 | 211 | 392 |
| Colored | 65 | 72 | 137 |
| Foreign | 65 | 58 | 123 |
| Totals | 311 | 341 | 652 |
| Still Birth | | | 70 |

JAMES A. STEUART, M. D.,
Commissioner of Health and Registrar.

By Order: A. R. CARTER,
Secretary.

U. S. SIGNAL OFFICE,
BALTIMORE, MD., February 1, 1880.

### METEOROLOGICAL SUMMARY FOR THE MONTH OF JANUARY, 1880.

| | | | |
|---|---|---|---|
| Mean Barometer . . . . . | 30.212 | inches | |
| Highest Barometer . . . . . | 30.703 | " | on the 29th |
| Lowest " . . . . . | 29.562 | " | on the 20th |
| Monthly Range of Barometer . . | 1.241 | " | |
| Mean Temperature . . . . . | 43.1 | degrees | |
| Highest Temperature . . . . . | 65 | " | on the 28th |
| Lowest " . . . . . | 17 | " | on the 14th |
| Monthly Range of Temperature . . | 48 | " | |
| Mean Daily Range of Temperature . | 14.3 | " | |
| Mean Relative Humidity . . . | 71.9 | per cent. | |
| Prevailing Wind—N. W. Highest Velocity | 20 | miles per hour | |
| Total Movement of Wind . . . | 3442 | " | |
| Total Rainfall, or Melted Snow . . | 2.26 | inches | |
| Total Number of Days on which Rain or Snow Fell . . . . . . | 17 | | |

NOTE.—The mean temperature of January, 1880, is eight degrees (8°) above the average January mean, and is the highest on record since the establishment of the Signal Office, ten years ago. The absence of high winds is another remarkable feature of the month.

ROBERT SEYBOTH,
Sergeant Signal Corps, U. S. A.

### A LETTER OF INDIVIDUAL IMPORTANCE TO EVERY ONE INTERESTED IN DENTISTRY.

DEAR DOCTOR:—I am preparing for publication "A Directory, History and Biography of Dentistry of the World," and am exceedingly anxious that your name and address should correctly appear in its pages. I have at this time gathered about 10,000 names, but fearing that there may be an error in your name or address, or that it may be omitted altogether, I *earnestly request* that you send me your printed or plainly written professional card, containing your full address, with degrees, titles and honors.

You will also please answer the following questions: What is your native tongue and with what languages are you conversant? Please state briefly: What colleges or institutions have you attended? Of what societies, if any, are you a member? What office, if any, have you held in either?

This work will contain a general history of dentistry and its individual history in each country, written by authors who are acknowledged authorities. It will also contain the biography of those of the profession who have been its greatest benefactors. There will be much other matter introduced which will make it a work of great value to the profession. It will be especially useful in having the address of all the dentists in the world. To this end I would request every dental college to send me its name, address, date of organization, number of teachers, with their duties, and all other matters of interest to the dental profession.

All dental societies and other organizations pertaining to dentistry, will please send their names, location, time of meeting, number of members, when organized, and all other important information, with the printed copy of the laws of their State and Country governing the practice of dentistry.

I ask the co-operation of all dental journals, colleges, societies, dealers and individuals interested in dentistry in all parts of the world, and any information not asked for in the above, which you may think of interest, will be thankfully received.

All dental manufacturers and dealers in the world will please send me their business cards immediately.

It will be evident to all that to complete a work like the one proposed, will require considerable time and money, both of which have already been largely expended.

I have secured copyrights for the book, and it will be sold by subscription. All subscribers who send in their money before November 1st, 1880, will have their names in **heavy letters**, which names will generally be selected as liberal persons, therefore to such will most likely be sent circulars, papers, books, etc., setting forth all that is new and useful to the dental profession. In order to secure your name in the Directory, you will please send the desired information *without delay*, and state whether you would like to subscribe.

The price of the book will be five dollars. Remember that you are not required to purchase it in order to have your name inserted.

All dental journals in the world will please copy this article for the good of the profession at large, and send me a copy. We will then give your journal ample notice in the Directory. Hoping that this will receive your favorable and prompt attention, I am,

Very respectfully,

B. M. Wilkerson, M. D., D. D. S.,
Junior Editor and Proprietor of "The Independent Practitioner,"
68 N. Charles Street, Baltimore, Md.,
United States of America.

